# Giant low-grade primary myofibroblastic sarcoma of the posterior chest wall

**DOI:** 10.1186/s12957-017-1167-7

**Published:** 2017-05-03

**Authors:** Darko Katalinic, Fedor Santek

**Affiliations:** 1 Department of Internal Medicine, Faculty of Medicine, J.J. Strossmayer University of Osijek, Cara Hadrijana 10/E, HR-31000 Osijek, Croatia; 2 Department of Oncology, Faculty of Medicine, University Hospital Centre Zagreb, Zagreb, Croatia

**Keywords:** Myofibroblastic sarcoma, Chest wall, Surgery, Chemotherapy

## Abstract

Primary myofibroblastic sarcoma is an extremely rare, highly malignant neoplasm, and only few cases had been reported in the literature worldwide. In the present study, we report an unusual case of a low-grade myofibroblastic sarcoma located in the posterior chest wall with intrathoracic propagation and discuss its clinical and pathological features.

## To the Editor,

Myofibroblastic sarcoma (MS) is considered as an extremely rare entity of soft tissue neoplasms, defined as a malignant neoplastic myofibroblastic proliferation with fibromatosis-like features [1]. It has been known to arise mainly at the head and neck regions, although it could be rarely found at the extremities and trunk, and thus, only a small number of cases have been reported in the literature worldwide [1, 2]. Treatment primarily involves surgical resection while the role of adjuvant or palliative radio-chemotherapy remains unclear [2]. We would like to draw attention to a case of low-grade myofibroblastic sarcoma (LGMS) occurring in the posterior chest wall of a 41-year-old man and discuss its clinical, radiological and microscopical features.

**Fig. 1:**
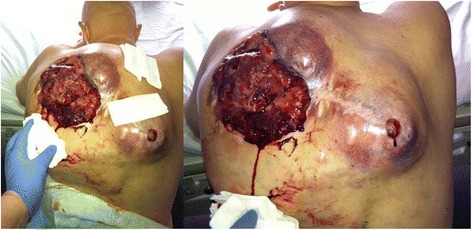
Clinical picture of the exulcerated bleeding myofibrosrcoma of the posterior chest wall

**Fig. 2:**
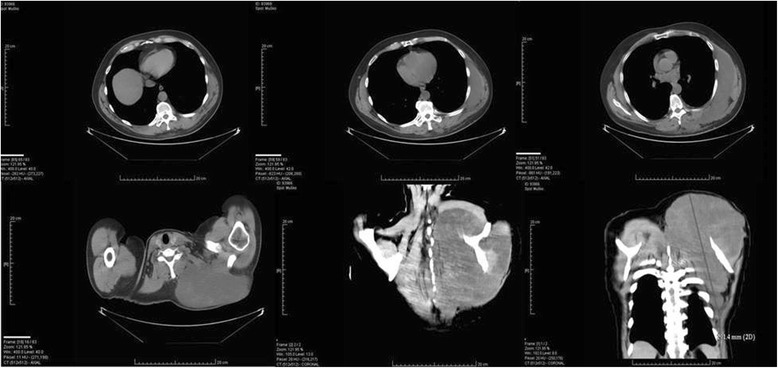
Computed tomography imaging of the chest showing giant tumor mass measuring 29 × 22 cm in size with destruction of the chest wall and propagation in to the chest cavity

**Fig. 3:**
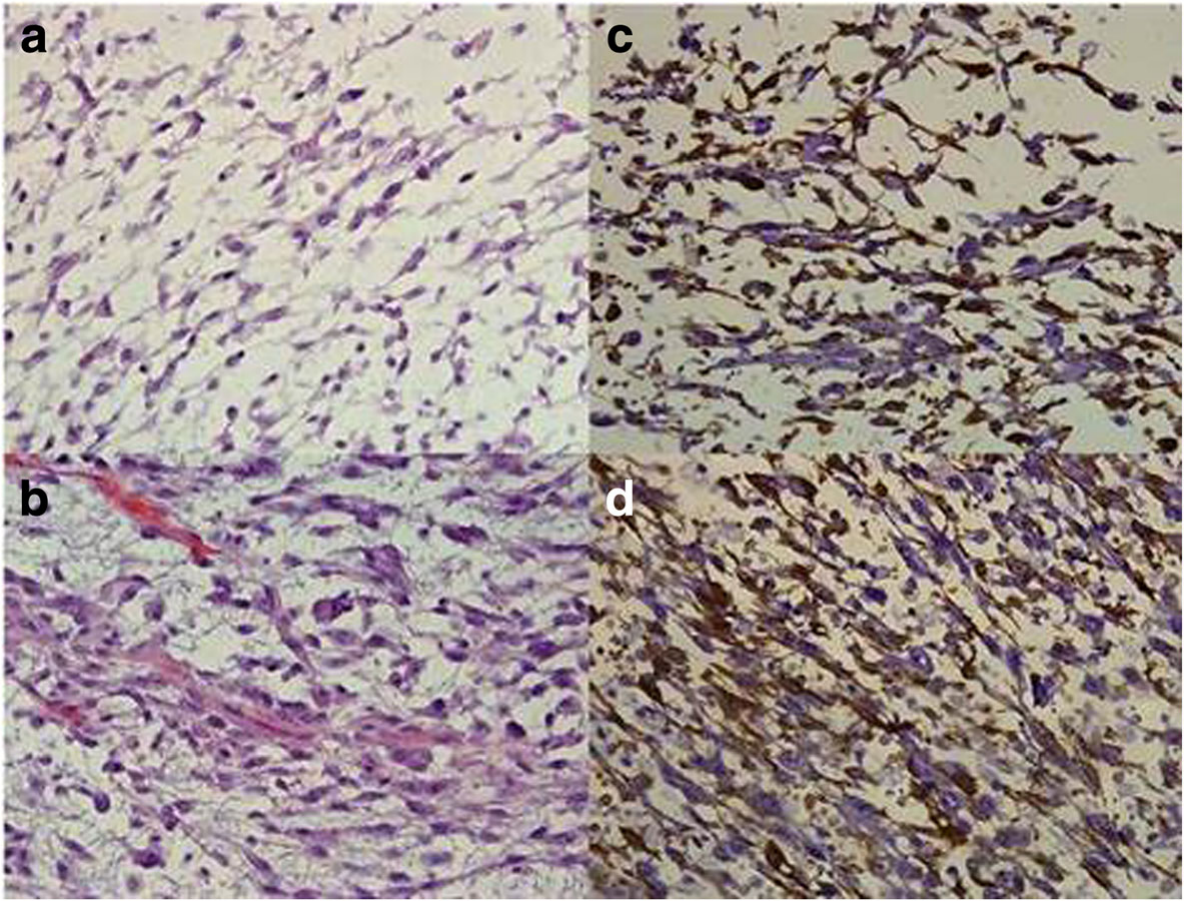
Histopathological analysis showing spindle-shaped tumour cells arranged in interlacing fascicles or storiform pattern with scattered necrosis and haemorrhage (**a**–**b**; HE staging, original magnification ×400). The tumour cells were positive for SMA and CD99 and negative for desmin and CD34, all consistent with low-grade myofibrosarcoma (**c–d**; immunohistochemical staging, original magnification ×400)

A 41-year-old man was referred for the management of painful bleeding and swelling in the area of the left scapula (Fig. 1). A giant bleeding tumour, 29 × 22 cm in size, had been growing insidiously for the preceding 2 months before hospital admission (Fig. 2). The mass was surgically removed with early relapse within several months. The histopathology examination showed malignant SMA+, Desmin−, CD99+, S100+/ALK−, and CD34− sarcoma cells with nuclear hyperchromasia and mild cellular pleomorphism. The histological features were compatible with a very rare entity—the LGMS (Fig. 3). The findings of further metastatic workups were positive. The patient received adjuvant chemotherapy (6× MAID and 2× ICE regimen) with significant clinical improvement, and 6 months progression-free survival was achieved.

MS is a rare infiltrative low- or high-grade mesenchymal tumour that arises usually in a soft tissue with a predilection for the head and neck region. Other locations on the body are extremely rare [1, 2]. While MS has indolent course, in our case, the disease presentation were very specific with the pain and tumour growth in the region involved. The radiological features of MS have not been well documented because of its rareness. Nevertheless, computed tomography, magnetic resonance imaging and positron emission tomography are effective methods in detecting the tumour, esspecially by developing in the thoracic or abdominal cavity [3]. The exact diagnosis of MS relies on pathological findings [3–5]. The diagnosis must be differentiated from malignant fibrous histiocytoma, leiomyosarcoma, inflammatory myofibroblastic tumour and other various reactive conditions or benign tumours. Histopathologically and immunohistochemically, LGMS is composed of slender spindle cells with vesicular nuclei showing immunopositivity for CD99, vimentin and SMA, while desmin and ALK are mostly immunonegative [3–5]. The electron microscopy as well as cytogenetic and molecular genetic alterations of the tumours are presently obscure, and they need to be reviewed in the future. Clinically, LGMS is an aggressive neoplasm with frequent recurrence rate and high metastatic potential and should be treated by surgicaly excision. The surgery should be performed by an experienced surgeon with preserving the function of blood vessels, muscles and nerves where possible. Evaluation of the resectability of a LGMS depends on the tumour stage and the patient’s co-morbidity. The growth pattern of the cancer requires that a wide margin of normal tissues be removed with tumour debulking. This requirement, due to the anatomical condsiderations, must determine the type of operation. An acceptable margin of normal tissue is commonly accepted as 1 cm soft tissue. If wide resection is not possible, it may be acceptable to leave microscopic-positive surgical margins but this cases require adjuvant treament with radio-chemotherapy [6]. Only under these conditions, a favourable prognosis may be attained. The therapeutic effects of adjuvant chemotherapy or radiation therapy are still unclear [1, 2]. Our patient was treated with MAID and ICE regimen, and we were able to achive a 6-month progression-free survival. Preoperative diagnosis is based on clinical examination, imaging techniques and biopsy of suspected lesion as well as intraoperative histological diagnosis. The standard medical follow-up must include clinical examination to focus on local recurrence and radiological evaluation where indicated by clinical suspicion. Finally, the main clinical challenge remains prevention of local recurrence or metastatic spreading. Therefore, periodic imaging examinations and regular medical lifelong follow-up is mandatory [6].

## Abbrevations

ALK: Anaplastic lymphoma kinase
CD34: Cluster of differentiation 34
CD99: Cluster of differentiation 99
ICE: Ifosfamide, carboplatine, etoposide
LGMS: Low-grade myofibroblastic sarcoma
MAID: Mesna, doxorubicin, ifosfamid, dacarbazine
MS: Myofibroblastic sarcoma
S100: S100 protein
SMA: Smooth muscle actin
